# Multiple Transmission Chains within COVID-19 Cluster, Connecticut, USA, 2020^1^

**DOI:** 10.3201/eid2710.211196

**Published:** 2021-10

**Authors:** Stephen M. Bart, Eileen Flaherty, Tara Alpert, Sherry Carlson, Lisa Fasulo, Rebecca Earnest, Elizabeth B. White, Noel Dickens, Anderson F. Brito, Nathan D. Grubaugh, James L. Hadler, Lynn E. Sosa

**Affiliations:** Centers for Disease Control and Prevention, Atlanta, Georgia, USA (S.M. Bart);; Connecticut Department of Public Health, Hartford, Connecticut, USA (S.M. Bart, E. Flaherty, L.E. Sosa);; Yale School of Public Health, New Haven, Connecticut, USA (T. Alpert, R. Earnest, E.B. White, N. Dickens, A.F. Brito, N.D. Grubaugh, J.L. Hadler);; local health departments, Connecticut, USA (S. Carlson, L. Fasulo)

**Keywords:** COVID-19, cluster, school, fitness center, genomic epidemiology, respiratory infections, severe acute respiratory syndrome coronavirus 2, 2019 novel coronavirus disease, coronavirus disease, zoonoses, viruses, coronaviruses, SARS-CoV-2, Connecticut, United States, transmission, community transmission

## Abstract

In fall 2020, a coronavirus disease cluster comprising 16 cases occurred in Connecticut, USA. Epidemiologic and genomic evidence supported transmission among persons at a school and fitness center but not a workplace. The multiple transmission chains identified within this cluster highlight the necessity of a combined investigatory approach.

During widespread community transmission of severe acute respiratory syndrome coronavirus 2 (SARS-CoV-2), transmission chains are sometimes unclear. Although often unavailable, viral genome sequencing can complement epidemiologic investigations.

In fall 2020, the Connecticut Department of Public Health analyzed data from contact tracing interviews and initially identified 5 cases of coronavirus disease (COVID-19), the illness caused by SARS-CoV-2, in employees of a single workplace within 1 week. One employee also worked at an elementary school and fitness center; in those settings, several contacts of this employee later tested positive for SARS-CoV-2. At the time, the weekly community case rate in this county was 141 cases/100,000 persons (https://portal.ct.gov/Coronavirus/COVID-19-Data-Tracker), reflecting high community transmission according to thresholds set by the Centers for Disease Control and Prevention (CDC) (*1*). To better characterize this cluster, we investigated its scope, phylogenetic relationships, and factors associated with transmission.

## The Study

We defined a cluster-associated case as COVID-19 in a coworker, primary contact, or secondary contact of the initial 5 employees; all cases were diagnosed by a viral test (i.e., antigen or nucleic acid amplification tests) authorized for emergency use by the Food and Drug Administration (*2*). We defined the investigation period as starting 1 week before symptom onset of the earliest workplace case and ending 2 weeks after symptom onset of the last workplace case. We assessed symptoms, onset dates, adherence to prevention strategies, and potential exposures. This activity was reviewed by CDC and was conducted in accordance with applicable federal law and CDC policy (e.g. 45 C.F.R. part 46.102(l) [*2*], 21 C.F.R. part 56; 42 U.S.C. 241(d); 5 U.S.C. 552a; 44 U.S.C. 3501 et seq.).

We extracted SARS-CoV-2 RNA from clinical nasopharyngeal specimens and conducted genomic sequencing using an amplicon-based approach with the MinION (Oxford Nanopore Technologies, https://nanoporetech.com) (*3*). We reconstructed maximum-likelihood phylogenetic trees using IQ-Tree with a general time-reversible nucleotide substitution model (*4*) (Appendix 1; Appendix 2 Table). 

**Figure 1:**
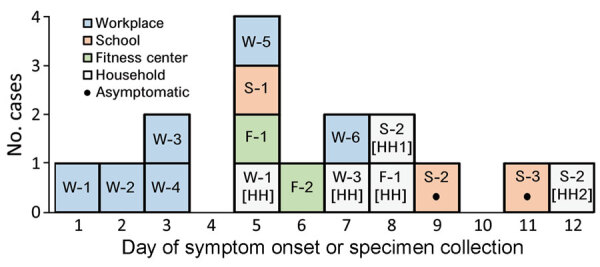
Epidemic curve for coronavirus disease cluster, Connecticut, USA, 2020. Symptomatic cases are plotted by day of symptom onset; asymptomatic cases are plotted by specimen collection day. Workplace employee W-3 had contacts in the school and fitness center. W, workplace; S, school; F, fitness center; [HH], household.

Overall, we identified 16 cluster-associated cases in 6 workplace employees, 3 school staff members and students, 2 fitness center attendees, and 5 household contacts. Symptom onset was generally earlier among workplace employees than among school and fitness center contacts (Figure 1).

The workplace employed 35 persons and provided in-person customer service. After the first employee (W-1) experienced symptoms on day 1 and tested positive for SARS-CoV-2, the workplace closed and recommended SARS-CoV-2 screening for other employees. In addition to the 5 initial cases, we identified 1 other case in a workplace employee (Figure 1). All 6 employees worked during the week before their symptoms began (Appendix 1 Figure 1).

In total, 4 of the 6 employees agreed to be interviewed (Appendix 1 Table 1). W-1 reported a potential exposure outside the workplace during the week before symptom onset. Two employees (W-2 and W-5) had contact with each other outside of work. No other employees reported contact with coworkers or members of coworkers’ households outside the workplace. Some employees were unable to maintain 6 feet of distance from coworkers and occasionally removed masks near coworkers. To increase air circulation, ventilation system fans were run continuously. Customers were not required to wear masks, and customer visits lasted 45–60 minutes.

One employee (W-3) also worked at an elementary school that offered in-person education 5 days a week. W-3 worked at the school on outbreak days 1–3; W-3’s symptoms developed on day 3. Three school contacts of W-3 subsequently tested positive for SARS-CoV-2 infection: a staff member (S-1) and 2 students (S-2 and S-3). S-1, a staff member, spent most of their time in a neighboring classroom but had brief contact with W-3 while substituting for W-3’s classroom. W-3 and S-1 reported strict adherence to prevention measures, including masking and social distancing, and did not have contact outside of school. To improve ventilation, the classroom windows were kept open. Among ≈15 students in W-3’s classroom, 2 asymptomatic students (S-2 and S-3) tested positive for SARS-CoV-2. S-2 was tested after a family member (S-2 [HH1]) had COVID-19 symptoms; another family member (S-2 [HH2]) later experienced symptoms as well. S-3 was tested after being notified that another person in the classroom tested positive for SARS-CoV-2.

W-3 taught an indoor fitness class on day 2, the day before their symptom onset. Approximately 6 clients attended the 1-hour class. Attendee F-1 experienced symptoms on day 5; attendee F-2 experienced symptoms on day 7. A household contact of F-2 (F-2 [HH]) later tested positive for SARS-CoV-2. W-3 and F-1 reported that attendees wore masks before and after the class but removed them during distanced (i.e., >6 feet) exercise. Information regarding facility ventilation was unavailable.

**Figure 2:**
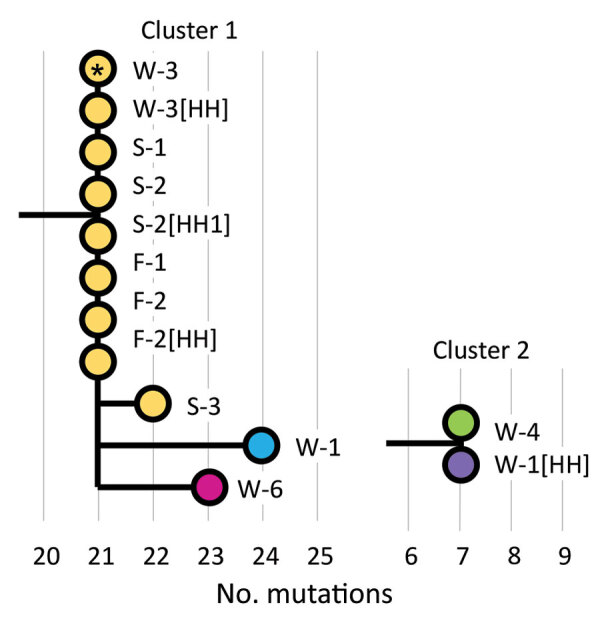
Maximum-likelihood phylogenetic tree for coronavirus disease cluster, Connecticut, USA, 2020. Wuhan/Hu-1/2019 (GISAID accession no. EPI_ISL_402125; https://www.gisaid.org) and Wuhan/WH01/2019 (accession no. EPI_ISL_406798) were used as reference genomes. Workplace employee W-3 (asterisk) had contacts in the school and fitness center. Colors correspond with presumed transmission chains based on epidemiologic and genomic data. W, workplace; S, school; F, fitness center; [HH], household.

We acquired 13 specimens for viral genome sequencing. Specimens were unavailable for 2 workplace employees (W-2 and W-5) and 1 student household contact (S-2 [HH2]). The resulting genomes clustered into 2 separate lineages (Appendix 1 Figure 2). Cluster 1 comprised 11 genomes, of which 9 were identical or differed by 1 mutation. These 9 genomes were extracted from samples from W-3, W-3’s household contact, the school staff and students, the fitness center attendees, and household contacts of persons at the school and fitness center (Figure 2). The other 2 genomes in cluster 1 were isolated from W-1 and W-6. W-1 was the only employee to work during the infectious period (defined as beginning 2 days before symptom onset); however, sequences for W-3 and W-6 differed from W-1’s sequence by >3 mutations. Cluster 2 comprised genomes isolated from a workplace employee and the household contact of another employee (Figure 2); there was no known epidemiologic link between these 2 persons.

## Conclusions

We found that the 16 members of a single COVID-19 cluster were involved in multiple transmission chains. Epidemiologic and genomic evidence supported transmission in the school and fitness center but not the workplace. These findings highlight challenges in accurate delineation of SARS-CoV-2 transmission chains and emphasize the benefits of combined epidemiologic and genomic investigation.

Although diagnostic specimens are often discarded by laboratories soon after testing, rapid identification of this cluster enabled the acquisition of specimens from 13 of the 16 cases. Our results suggest that infection was directly transmitted from W-3 to >6 other persons within their household, school, and fitness center. Classroom transmission of SARS-CoV-2 is uncommon in the context of prevention strategies such as masking and distancing; previous studies have suggested that most school-associated cases are acquired outside of school (*5*,*6*). However, our results suggest that staff-to-staff and staff-to-student transmission occurred in this classroom. This investigation also adds to evidence that indoor exercise without masks can facilitate SARS-CoV-2 transmission (*7*,*8*). Fitness centers might consider moving high-exertion exercise outdoors, improving ventilation, and promoting mask use during indoor exercise. Mask use during indoor exercise was mandated in Connecticut later in November 2020 (*9*).

Genomic data did not indicate SARS-CoV-2 transmission among workplace employees. Divergence among viral sequences of workplace employees and the SARS-CoV-2 evolutionary rate of ≈1 mutation per 2 weeks (*10*) suggest that the 4 other workplace cases were each acquired independently. However, workplace transmission from unidentified employees or customers remains possible. In addition, a workplace employee and household contact had unrelated sequences, suggesting that they also were infected independently (Figure 2). This apparent workplace cluster, disproven by sequencing, highlights challenges in defining transmission chains during widespread SARS-CoV-2 community transmission. These findings highlight the crucial role of genomic sequencing in clarifying transmission chains.

Appendix 1 Additional information on multiple transmission chains within COVID-19 cluster, Connecticut, USA, 2020.

Appendix 2 Additional genomes used in multiple transmission chains within COVID-19 cluster, Connecticut, USA, 2020.
