# Supplementary material for: Multiple Transmission Chains within COVID-19 Cluster, Connecticut, USA, 2020
Source: Emerg Infect Dis. 2021 Oct;27(10):2669–72. doi: 10.3201/eid2710.211196 (PMC8462310; doi:10.3201/eid2710.211196)
Supplement: Appendix 1 — Additional information on multiple transmission chains within COVID-19 cluster, Connecticut, USA, 2020. [file 21-1196-Techapp-s1.pdf]

Article DOI: https://doi.org/10.3201/eid2710.211196

Multiple Transmission Chains within COVID-19 Cluster, Connecticut, USA, 2020

Appendix 1

Appendix Methods

Genomic Sequencing

Nucleic acid was extracted from available original severe acute respiratory syndrome coronavirus 2 (SARS-CoV-2) diagnostic specimens (300 µL) using the MagMAX viral/pathogen nucleic acid isolation kit (Thermo Fisher Scientific, https://www.thermofisher.com). Sequencing libraries were prepared using the Ligation Sequencing Kit and the Oxford Nanopore Technologies Native Barcoding Expansion pack (https://nanoporetech.com) as described in the ARTIC Network protocol with V3 primers (*1*) with the following modifications: cDNA was generated with SuperScriptIV VILO Master Mix (Thermo Fisher Scientific), all amplicons were generated using 35 cycles of amplification, amplicons were normalized to 15 ng for each sample, end repair incubation time was increased to 25 minutes followed by an additional bead-based clean-up, and all clean-up steps used a ratio of 1:1 bead-to-sample ratio. Twenty-five ng of the final library was loaded on a MinION R9.4.1 flow cell. The ARTIC Network RAMPART application was used to monitor approximate genome coverage for each sample during sequencing (https://github.com/artic-network/rampart). Fast5 files were basecalled using the Guppy basecaller 4.4.0 fast model. Consensus genomes were generated using the ARTIC bioinformatic pipeline (https://artic.network/ncov-2019/ncov2019-bioinformatics-sop.html), which uses Nanopolish to call variants (*2*). A threshold of 20× coverage was required for each amplicon to be included in the consensus genome.

Multiple extraction controls were included for each RNA extraction batch and tested negative for SARS-CoV-2 RNA by reverse transcription PCR. No-template controls were introduced for each run at the cDNA synthesis and amplicon synthesis steps and were taken through the entire library preparation and sequencing protocol to detect any cross-contamination.

For each control in each run, <1,000 total reads were observed. A subset of reads in control samples aligned to the SARS-CoV-2 genome, but no position of the genome had >20 reads (enough data to influence the generation of a consensus genome).

Phylogenetic analysis

Consensus genomes were aligned using MAFFT within an augur pipeline (*3*,*4*). Sites near the 5′ and 3′ end of the genomes were masked alongside other problematic sites (*5*). The phylogenetic analysis dataset consisted of genomes from this study along with 570 globally representative genomes from GISAID (https://www.gisaid.org; Appendix Table 2). The tree was rooted using 2 genomes from early periods of the pandemic: Wuhan/Hu-1/2019 (GISAID accession no. EPI_ISL_402125) and Wuhan/WH01/2019 (GISAID accession no. EPI_ISL_406798).

References

1. Quick J. nCoV-2019 sequencing protocol v3 (LoCost) V.3. 2020 [cited 2021 Mar 24]. https://www.protocols.io/view/ncov-2019-sequencing-protocol-v3-locost-bh42j8ye

2. Loman NJ, Quick J, Simpson JT. A complete bacterial genome assembled de novo using only nanopore sequencing data. Nat Methods. 2015;12:733–5. PubMed https://doi.org/10.1038/nmeth.3444

3. Katoh K, Standley DM. MAFFT multiple sequence alignment software version 7: improvements in performance and usability. Mol Biol Evol. 2013;30:772–80. PubMed https://doi.org/10.1093/molbev/mst010

4. Hadfield J, Megill C, Bell SM, Huddleston J, Potter B, Callender C, et al. Nextstrain: real-time tracking of pathogen evolution. Bioinformatics. 2018;34:4121–3. PubMed https://doi.org/10.1093/bioinformatics/bty407

5. De Maio N, Walker C, Borges R, Weilguny L, Slodkowicz G, Goldman N. Masking strategies for SARS-CoV-2 alignments. 2020 [cited 2021 Apr 30]. https://virological.org/t/masking-strategies-for-sars-cov-2-alignments/480

Appendix Table. Symptoms of persons in cluster of coronavirus disease, Connecticut, USA, 2020

Person	Fever or chills	Cough	Shortness of breath/difficulty breathing	Fatigue	Muscle or body aches	Headache	Loss of taste or smell	Sore throat	Congestion or runny nose	Nausea or vomiting	Diarrhea
W-1	X	X		X	X	X	X		X		
W-1 [HH]							X				
W-2	X	X	X	X			X	X			X
W-3		X	X	X		X			X		X
W-4		X	X			X		X	X		
S-1	X	X		X	X	X		X	X	X	
F-2	X	X	X	X	X	X	X		X	X	

*F, fitness center; HH, household member; Pt, patient; S, school; W, workplace employee.

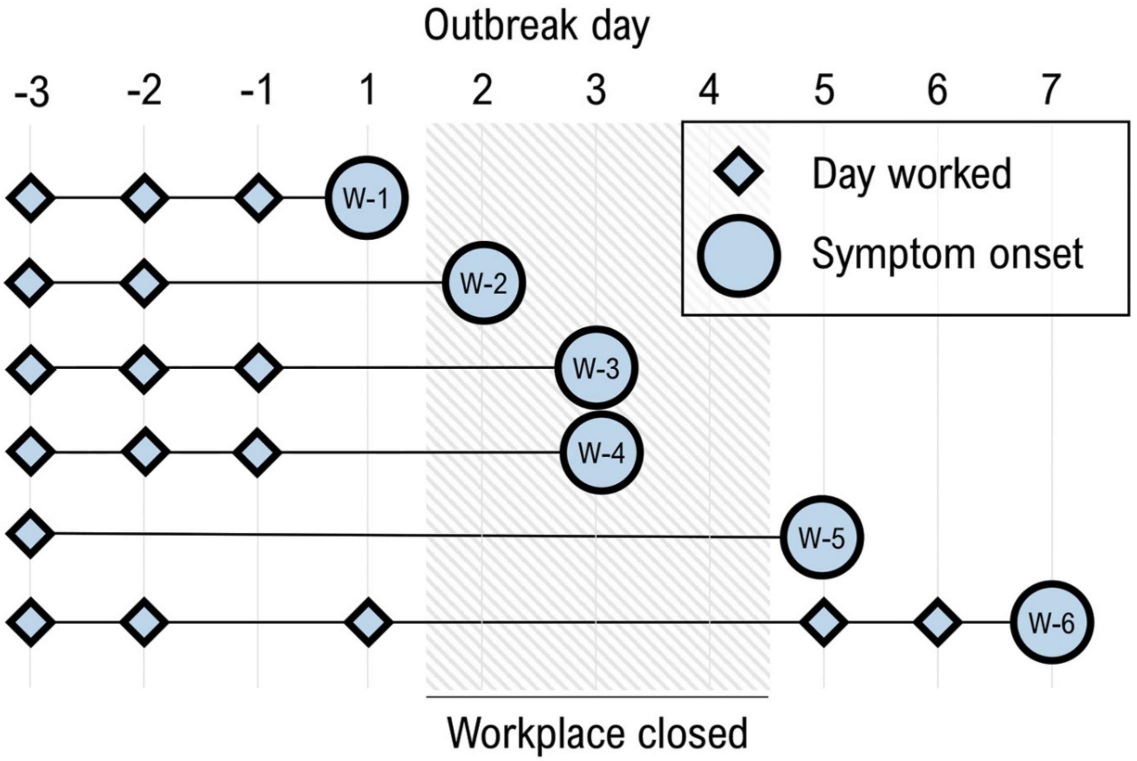

Appendix Figure 1. Employee work schedule and dates of symptom onset of patients in coronavirus disease cluster, Connecticut, USA, 2020. Diamonds indicate days worked according to workplace schedules and case investigation interviews; circles indicate reported symptom onset days for each case. No employees worked on the day of symptom onset. The workplace was closed on days 2–4.

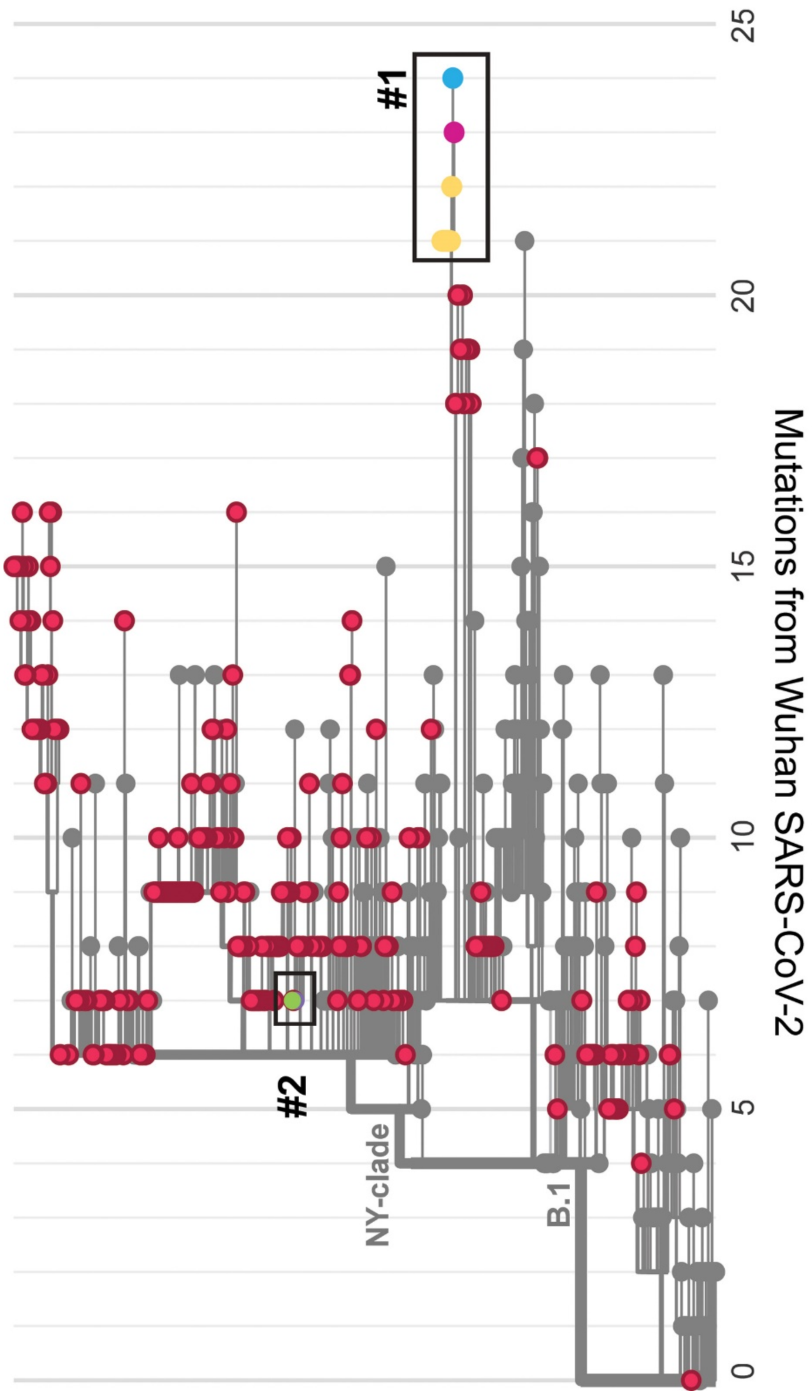

Appendix Figure 2. Maximum-likelihood phylogenetic tree for coronavirus disease cluster, Connecticut, USA, 2020 in comparison with international samples. Colors correspond with presumed transmission chains based on epidemiologic and genomic data. Circles indicate genomes; red indicates genomes from Connecticut. Boxes indicate Clusters 1 and 2.
